# Determination of Heavy Metals in Herbal Food Supplements using Bismuth/Multi-walled Carbon Nanotubes/Nafion modified Graphite Electrodes sourced from Waste Batteries

**DOI:** 10.1038/s41598-019-54589-x

**Published:** 2019-12-06

**Authors:** Shirley Palisoc, Remuel Isaac M. Vitto, Michelle Natividad

**Affiliations:** 1 Condensed Matter Physics Laboratory, De La Salle University, Manila, 922 Philippines; 2 Condensed Matter Research Unit, CENSER, De La Salle University, 2401 Taft Avenue, Manila, 922 Philippines

**Keywords:** Chemistry, Analytical chemistry, Sensors

## Abstract

An electrochemical sensor based on graphite electrode extracted from waste zinc-carbon battery is developed. The graphite electrode was modified with bismuth nanoparticles (BiNP), multi-walled carbon nanotubes (MWCNT) and Nafion via the drop coating method. The bare and modified graphite electrodes were used as the working electrode in anodic stripping voltammetry for the determination of trace amounts of cadmium (Cd^2+^) and lead (Pb^2+^). The modified electrode exhibited excellent electroanalytical performance for heavy metal detection in comparison with the bare graphite electrode. The linear concentration range from 5 parts per billion (ppb) to 1000 ppb (R^2^ = 0.996), as well as detection limits of 1.06 ppb for Cd^2+^ and 0.72 ppb for Pb^2+^ were obtained at optimized experimental conditions and parameters. The sensor was successfully utilized for the quantification of Cd^2+^ and Pb^2+^ in herbal food supplement samples with good agreement to the results obtained by atomic absorption spectroscopy. Thus, the BiNP/MWCNT/Nafion modified graphite electrode is a cost-effective and environment-friendly sensor for monitoring heavy metal contamination.

## Introduction

Heavy metal pollution is mainly a result of mining and smelting operations, industrial processes, and the use of metal compounds in domestic and agricultural activities^1,2^. To prevent exposure of humans and animals to these toxic elements, the World Health Organization (WHO) regulates the concentrations of trace heavy metals in water, soil, plants, food, and many more. The heavy metal intake for medicinal plants, for instance, was set by WHO with the permissible limit of 0.3 parts per million (ppm) for cadmium and 10 ppm for lead^3^. Due to the adverse effects of heavy metals on human health and on the environment, the determination of heavy metals has attracted considerable attention in recent years^4–17^. Analytical techniques such as atomic absorption spectrometry, laser-induced plasma spectroscopy, inductively coupled plasma-mass spectrometry, colorimetric analysis, high-performance liquid chromatography^18–22^, and electrochemical methods such as anodic stripping voltammetry (ASV) have been developed to detect trace amounts of heavy metals in samples^23–26^. Chromatographic and spectrophotometric methods, however, are not an ideal technique because they are expensive, they require complex process and instruments, and are time-consuming. On the other hand, electrochemical method is a fast and efficient way of determining the concentrations of heavy metals in samples due to its simplicity, high sensitivity, good stability, and low cost.

The glassy carbon electrode (GCE) is commonly used as the working electrode in voltammetry due to its high sensitivity and selectivity^27^. Nonetheless, the excellent properties of GCE come in exchange for its high cost^28^. Alternatively, graphite electrodes (GE) are ideal for the electrochemical analysis of heavy metals as they offer attractive features, such as, good electrical conductivity, low background current, and little or no pretreatment is required^29^. Graphite can be easily obtained because it is abundantly available in waste dry-cell batteries. Eveready India Ltd. is the world’s third largest producer of carbon zinc batteries which sells more than a billion units per year and with a projected increase in net sales and operating profit for the years 2018 and 2019^30^. Thus, access to recycled batteries will not be a problem in the coming years. Moreover, the graphite rods extracted from waste AA batteries have ideal measurements (2 mm or 3 mm in diameter) that fit the fabrication of a good working electrode^31^. However, bare graphite electrodes have a certain disadvantage when used in electrochemical analysis as they usually have a low sensitivity due to their high activation overpotential^32^. The structure of graphite is comprised of sp2 hybridized carbon atoms, arranged in a graphene layer in a honeycomb lattice structure, and each layer has a free valence electron that enables bonding with other graphene layers and other elements through the Van der Waals interaction^33^. Due to this unique structure of graphite, its surface can be easily modified with various elements to increase its sensitivity.

Different materials have been employed for the modification of electrodes used in electrochemical analysis^34–48^. Bismuth nanoparticles (BiNP) have become one of the most popular electrode modifiers due to its broad electrochemical window and low toxicity. Studies have shown that modification of the working electrode with bismuth has effectively improved the electrode’s analytical performance in ASV compared to a bare or unmodified electrode^49,50^. Modification with multi-walled carbon nanotubes (MWCNT) has also been reported to increase the electrode’s electrical conductivity, decrease the probability of surface fouling^51^, and increase the rate of electrochemical reactions^52^. Nafion has also been widely used as an electrode modifier because it is a perfluorinated sulphonated cation exchanger and it has excellent properties such as antifouling capacity, chemical inertness, and high permeability to cations^53,54^. Hence, the combination of BiNP, MWCNT, and Nafion as electrode modifiers would bring a synergistic effect that would greatly increase the selectivity and sensitivity of the electrode.

Herbal food supplements (HFS) continue to become more and more popular worldwide with a significant population turning into these products for the treatment of various health problems and/or for the augmentation of deficiencies in vitamins and minerals^55^. However, concerns in public health issues regarding their safety have arisen as many of them have not yet been tested and are poorly monitored. The safety is often conflicted by insufficient control in their quality, incomplete labeling, and the absence of proper user information^56^. Thus, their known possible adverse effects are very limited and it is difficult to determine the safest and effective way of using them. Moreover, it has been reported in some studies that some HFS contain heavy metals^57,58^. It is therefore important to determine possible heavy metal contamination in HFS.

The aim of this study is to develop a cost-effective and environment-friendly electrochemical sensor based on graphite rod extracted from waste zinc-carbon battery. The extracted rods were modified with BiNP, MWCNT and Nafion and were used as the working electrode in ASV to detect trace amounts of cadmium (Cd^2+^) and lead (Pb^2+^). To demonstrate the utility of the modified electrodes in sensing applications, real sample analysis using the modified graphite electrode was done on commercially available herbal food supplements.

## Methodology

### Materials and equipment

Bismuth nanopowder was procured from Luoyang Tongrun Info Technology Co., Ltd. (Luoyang City, Henan, China). Multi-walled carbon nanotubes were procured from Cheap Tubes Inc. (Cambridgeport, Vermont, USA), and Nafion solution was procured from Fuel Cell Earth (Woburn, Massachusetts, USA). Propanol, ethanol, and hydrochloric acid were procured from RCI Labscan Ltd. (Bangkok, Thailand). Cadmium chloride and lead chloride were procured from Sigma-Aldrich (Singapore) and sodium chloride was procured from Techno Pharmchem (Barakhamba, Delhi, India). A BOSCH SAE200 electronic balance (BOSCH-Wägesysteme GmbH, Jungingen, Germany) was used to measure the amounts of bismuth nanopowder, MWCNT, cadmium chloride, lead chloride, and sodium chloride. A BANDELIN SONOREX sonicator (BANDELIN electronic GmbH & Co. KG, Berlin, Germany) was used to clean the glassware and electrodes. The coating solutions were homogenized using a Misonix ultrasonicator (Misonix, Inc., Farmingdale, New York, USA). A THERMOLYNE 4800 furnace (Barnstead International, Dubuque, Iowa, USA) was used for drying the graphite rods and dry-ashing of the real samples. An Autolab PGSTAT128N potentiostat (MTI Corporation, Richmond, CA, USA) was utilized for the ASV measurements and an AA-6300 Shimadzu atomic absorption spectrophotometer (Shimadzu, Tokyo, Japan) was used for atomic absorption spectroscopy (AAS) analyses. A JSM-5310 JEOL scanning electrode microscope coupled with energy dispersive X-ray spectroscopy (JEOL USA Inc., Peabody, Massachusetts, USA) was used for the surface morphology and elemental analyses. A BTX-285 × -ray powder diffractometer was used in the crystal structure analysis, and a Fourier transform-infrared spectrometer was utilized for the identification of the chemical components.

### Preparation of graphite electrodes

The graphite rods were extracted from different commercial brands of waste zinc-carbon batteries by removing the steel casing, top and bottom terminals, paper gasket, washer, wrapper, manganese dioxide powder and zinc pieces surrounding the graphite rod^59^. The extracted rod was cleaned by submerging it in propanol and sonicating it for 20 minutes. Once the graphite was cleaned, it was dried in a furnace at 150 °C for 48 hours. After drying, the tip of the electrode was polished with silicon-carbide sandpaper starting with a grade of 400 and all the way up to 5000. It was then polished with 0.3 µm followed by 0.05 µm alumina slurry on a glass slide. After the electrode has been polished, it was then cleaned by sonicating it for 5 minutes in ethanol, followed by deionized water and was then wrapped with Teflon tape in order to insulate the lateral exposure of the rod. The fabricated graphite electrode was kept in a desiccator at room temperature until further use.

### Fabrication of modified graphite electrodes

For the preparation of BiNP-MWCNT-Nafion suspension, BiNPs and MWCNTs were weighed and were subsequently mixed with a 1% (v/v) Nafion made of 0.333 ml 15% (v/v) Nafion and 4.667 ml ethanol solution. The amounts of BiNP and MWCNT in the suspension were each varied at 0.5 mg, 1.0 mg, and 1.5 mg to determine the best combination of modifiers. The mixtures were then ultrasonicated for 2 hours. Finally, the graphite electrode was drop-coated with the BiNP-MWCNT-Nafion suspension with the use of a micropipette, and the ethanol was allowed to evaporate at room temperature in ambient air.

### Preparation of stock solutions

Analyte solutions containing 0.1 M NaCl and 10 ppm of Cd and Pb were used for the optimization of the ASV parameters. This was done by adding 1.6 mg of CdCl_2_, 1.3 mg of PbCl_2_, and 0.5844 g of NaCl in 100 mL of deionized water. Ten (10) ppm solutions of Cd and Pb were used to obtain concentrations of 5 ppb, 7 ppb, 15 ppb, and the range from 100 to 1000 ppb for the calibration curves. The diluted solutions were then added with 0.5844 g of NaCl. All the resulting solutions were sonicated for 15 minutes.

### Real sample analysis

Three samples of commercially available herbal food supplements were procured from Quiapo District, Manila. The herbal food supplements were weighed obtaining 4 g for each sample in a 50 mL quartz crucible. The samples were then prepared for analysis through the modified dry-ashing and acid digestion techniques^60^. The samples were dry-ashed by heating in an oven for 12 hours at 500 °C and acid digested by dissolving them in 5 mL of 2.0 mol/L of HCl and heated in order to evaporate the solution until dry. The residue was filtered and diluted to 200 mL with deionized water and sonicated for 1 hour. Finally, it was divided into two equal parts, yielding 100 mL solution for ASV analysis and another 100 mL for AAS analysis.

### Anodic stripping voltammetry

Anodic stripping voltammetry was used to detect Cd^2+^ and Pb^2+^ in the prepared analyte solutions. The bare and modified graphite electrodes were used as the working electrode, an Ag/AgCl electrode as the reference electrode, and a platinum wire as the counter electrode. The ASV parameters were optimized by varying the initial potential, deposition time and scan rate. The calibration curves were obtained by varying the concentrations of Cd and Pb in the electrolyte solution.

**Figure 1:**
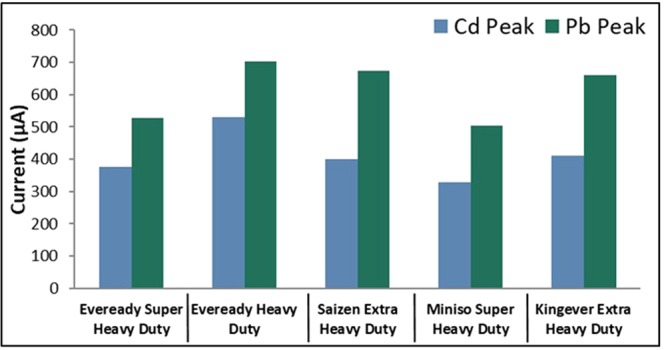
Anodic current peaks of graphite rods from different battery brands.

## Results and Discussion

### Determination of the best brand of graphite electrode

Five brands of commercial batteries were used in the simultaneous detection of 10 ppm each of cadmium and lead via ASV. The following ASV parameters were held constant: a cleaning step at +1 V for 60 seconds, a deposition potential of −0.9 V for 90 seconds, a rest period for 60 seconds, and a scan rate of 0.1 V/s. Figure 1 shows that the Eveready Heavy Duty brand resulted with the highest anodic current peaks; hence, it was considered as the best brand for the detection of Cd and Pb in this study. The electrode was further optimized by determining which polishing step will result in the highest anodic current peaks for Cd and Pb by comparing the effects of different grades of sandpaper and alumina slurry. The sandpaper grade of the final polish was varied at 2000-, 5000-, and 7000-grit and unpolished electrodes and electrodes polished with 0.3 µm and 0.05 µm alumina slurry were studied. The electrode polished with the 5000-grit sandpaper, 0.3 µm and 0.05 µm alumina slurry resulted in a higher anodic current peak.

**Figure 2:**
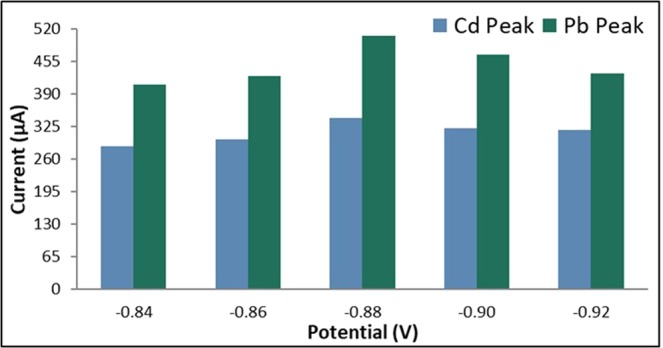
Anodic current peaks of bare GE for varying initial potential.

### Optimization of anodic stripping voltammetry parameters for bare graphite electrode

#### Initial potential

The applied initial potential was varied at −0.84 V, −0.86 V, −0.88 V, −0.90 V, and −0.92 V. The deposition time was held constant at 90 s, the rest period at 60 s, and the scan rate at 0.1 V/s. Figure 2 shows that the anodic current peaks for both Cd and Pb increased as the initial potential was decreased from −0.84 V to −0.88 V, while a significant decrease in the response of the anodic current peaks were observed when the initial potential reached a potential more negative than −0.88 V. This phenomenon could be ascribed to the evolution of hydrogen ions as the initial potential became more negative^61^. Therefore, the potential of −0.88 V was considered to be the optimum initial potential in this study.

#### Deposition time

**Figure 3:**
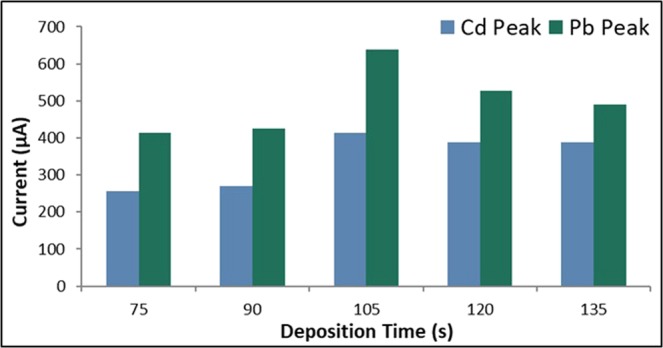
Anodic current peaks of bare GE for varying deposition time.

The applied deposition time was varied at 75 s, 90 s, 105 s, 120 s, and 135 s. The rest period was held constant at 60 s, the scan rate at 0.1 V/s, and the initial potential at −0.88 V. It can be observed in Fig. 3 that an increase in the current peaks for both Cd and Pb occurred as the deposition time was increased from 75 s to 105 s. This is due to the increase in the amounts of analytes adsorbed on the surface of the electrode as the deposition time was increased. However, a significant drop in the anodic current peaks was observed when the deposition time was further increased which can be attributed to the saturation of the electrode’s surface with Cd and Pb ions^62^. Therefore, the optimum deposition time was determined to be 105 s.

#### Scan rate

**Figure 4:**
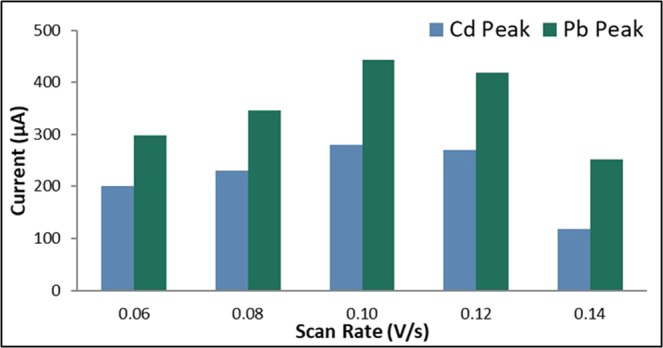
Anodic current peaks of bare GE for varying scan rate.

The applied scan rate was varied at 0.06 V/s, 0.08 V/s, 0.10 V/s, 0.12 V/s, and 0.14 V/s. The rest period was held constant at 60 s, the initial potential at −0.88 V and the deposition time at 105 s. Figure 4 shows that the anodic current peaks for both Cd and Pb increased as the scan rate was increased from 0.06 V/s to 0.10 V/s. Increasing the scan rate further resulted in a decrease in the current peaks. This is due to the heavy metal cations that readily form a cationic complex which is adsorbed by the Nafion cation-exchanger film. At higher scan rates, the complex cations are not completely reduced resulting to a lower current peak^63^. The best scan rate, therefore, was determined to be 0.10 V/s.

### Calibration curves for bare graphite electrode

**Figure 5:**
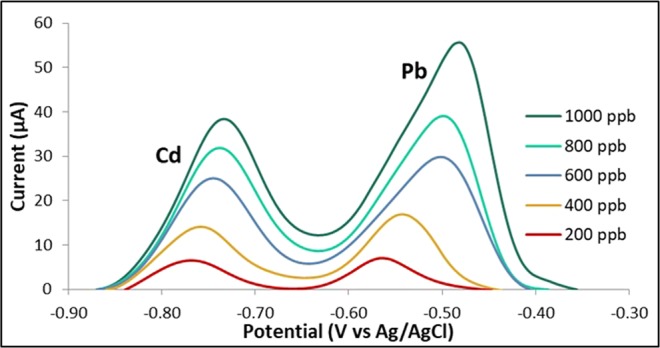
Voltammograms of bare GE for varying Cd and Pb concentrations.

**Figure 6:**
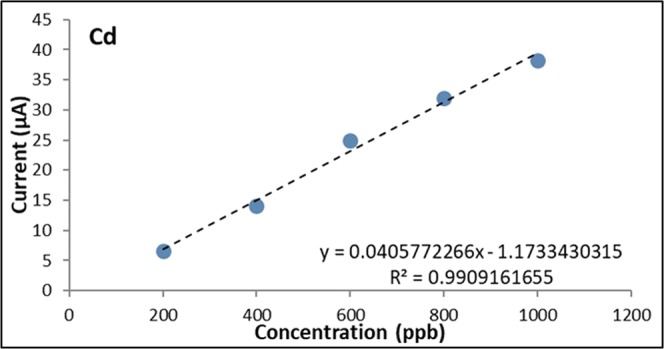
Calibration curve of bare GE for Cd.

**Figure 7:**
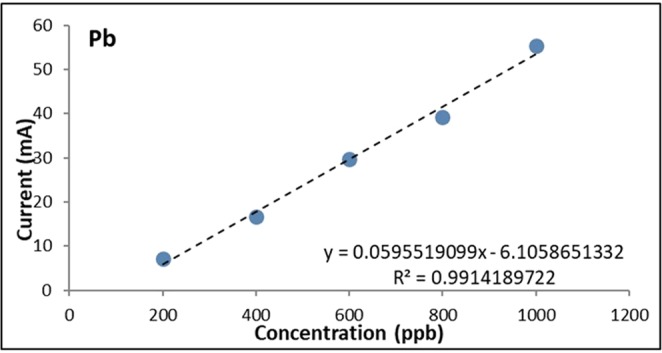
Calibration curve of bare GE for Pb.

The calibration curves were obtained by varying the concentrations of Cd and Pb from 100 ppb to 1000 ppb (Figs. 5, 6 and 7). The calculated values for R2 were found to be 0.9901 for Cd and 0.9914 for Pb which indicate a strong linear relationship between the heavy metal concentration and the anodic current peak.

### Optimization of modified graphite electrode

**Figure 8:**
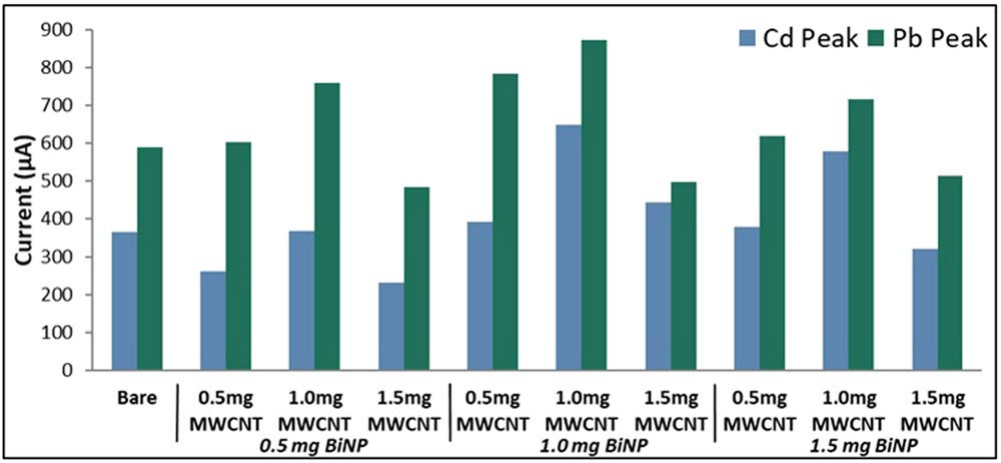
Anodic current peaks for varying amounts of modifiers.

The amount of BiNP was varied at 0.5 mg, 1.0 mg, and 1.5 mg while that of MWCNT was varied at 0.5 mg, 1.0 mg, and 1.5 mg. The concentration of the Nafion solution containing 0.333 mL of 15 wt.% Nafion and 4.667 mL of laboratory-grade ethanol was held constant. The resulting modified electrodes were used in the simultaneous detection of 10 ppm each of cadmium and lead via ASV. The following ASV parameters were held constant: a cleaning step of + 1 V for 60 s, an initial potential of −0.88 V, deposition time of 105 s, a rest period for 60 s, and a scan rate of 0.1 V/s. The anodic current peaks showed an increasing trend as the amounts of BiNP and MWCNT were increased from 0.5 mg to 1.0 mg as seen in Fig. 8. However, a significant decrease in the anodic current peaks for both heavy metals was observed as the amounts of BiNP and MWCNT were further increased further which can be ascribed to the oversaturation of the modifiers on the electrode surface. This resulted in a thick layer of the modifiers on the electrode surface which consequently suppressed its conducting capabilities. Since the electrode modified with 1.0 mg of BiNP and MWCNT exhibited the highest anodic current peaks for both Cd and Pb, it was chosen to be the best modified electrode in this study. The surface modification of the electrode significantly increased the anodic peaks for both heavy metals as compared to the bare electrode, as seen in Fig. 8. This can be attributed to the synergetic effects of BiNP, MWCNT and Nafion. The high conductivity and surface area-to-volume ratio of BiNPs and MWCNT increased the number of active sites within the electrode resulting to an increase in the amount of metal ions deposited on the electrode’s surface while the antifouling and cationic exchange capabilities of Nafion improved the stability of the electrode. In addition, for any amount of BiNP, the anodic current peaks for both Cd and Pb appear to be the highest when the amount of MWCNT is 1.0 mg, followed by 0.5 mg, and the lowest at 1.5 mg, except for the electrode modified with 1.0 mg BiNP and 0.5 mg MWCNT.

### Optimization of anodic stripping voltammetry parameters for modified graphite electrode

#### Initial potential

**Figure 9:**
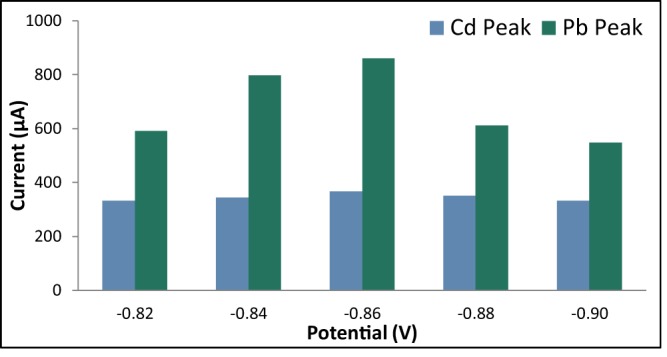
Anodic current peaks of modified GE for varying initial potential.

**Figure 10:**
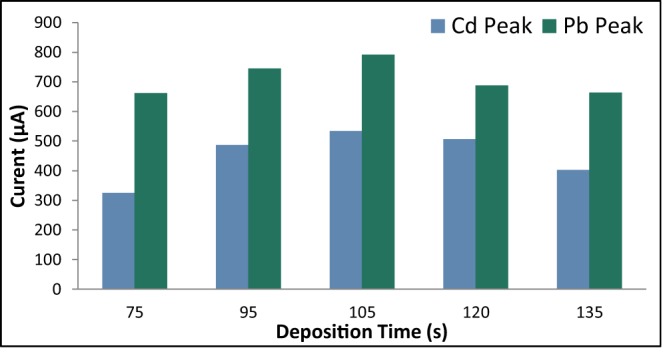
Anodic current peaks of modified GE for varying deposition time.

The initial potential was varied at −0.82 V, −0.84 V, −0.86 V, −0.88 V, and −0.90 V while the deposition time was held constant at 105 s, the rest period at 60 s, and the scan rate at 0.1 V/s. The anodic current peaks for Pb was observed to increase as the initial potential was decreased from −0.82 V to −0.86 V and decreased as the initial potential became more negative (Fig. 9). Hence,the initial potential of −0.86 V was considered to be the optimum potential. It can also be observed from Fig. 10 that the current peak for Cd did not vary much as the initial potential was varied. This is probably due to the preferred sensitivity of bismuth to ${{\rm{Pb}}}^{{2}^{+}}$ since the electronegativity of lead does not differ significantly from that of bismuth as compared to the difference between the electronegativities of cadmium and bismuth^64^.

#### Deposition time

The deposition time was varied at 75 s, 90 s, 105 s, 120 s, and 135 s. The other ASV parameters were held constant where the rest period was 60 s and the scan rate was 0.1 V/s and the initial potential was −0.86 V since its optimal value was already determined. An increase in the current peaks of Cd and Pb was observed as the deposition time was increased from 75 s to 105 s (Fig. 10). However, a deposition time of more than 105 s significantly decreases the current response peaks of Cd and Pb. Again, this is due to the huge amounts of the analytes that oversaturates the electrode’s surface^38^. A shift in the reduction potentials of Pb and Cd to a more cathodic potential was visible in the voltammograms, which was due to the increased amounts of the analytes which resulted to a higher potential needed to reduce the analytes^40^. Therefore, the optimal deposition time was determined to be 105 s.

#### Scan rate

**Figure 11:**
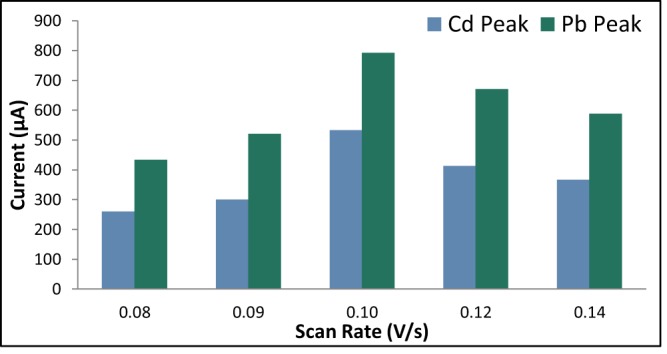
Anodic current peaks of modified GE for varying scan rate.

The scan rate was varied at 0.06 V/s, 0.08 V/s, 0.10 V/s, 0.12 V/s, and 0.14 V/s. The rest period was held constant at 60 s, the initial potential at −0.86 V, and the deposition time at 105 s. Figure 11 suggests that the optimum scan rate is 0.10 V/s. The shift in the reduction potentials is due to the fact that as the scan rate decreases, the analytes diffuses farther from the electrode surface. As a result, the reduction of Cd^2+^ and Pb^2+^ requires a more negative potential^65^.

### Calibration curves for the optimized modified graphite electrode

**Figure 12:**
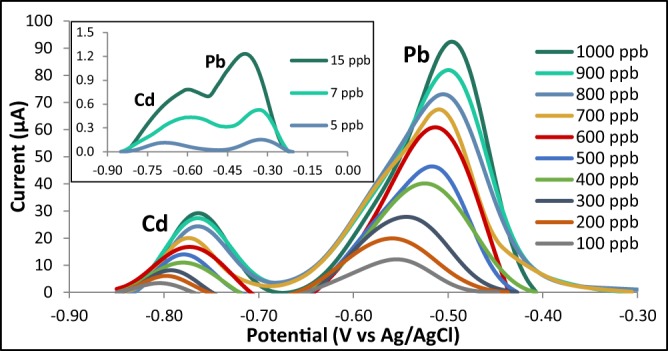
Voltammograms of the optimized modified GE for with varying analyte concentrations.

**Figure 13:**
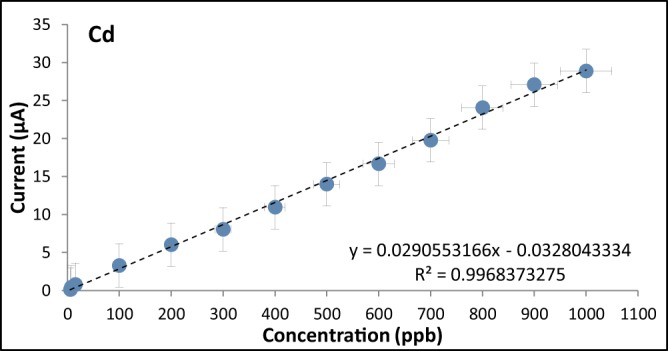
Calibration Curve of the optimized modified GE for Cd.

**Figure 14:**
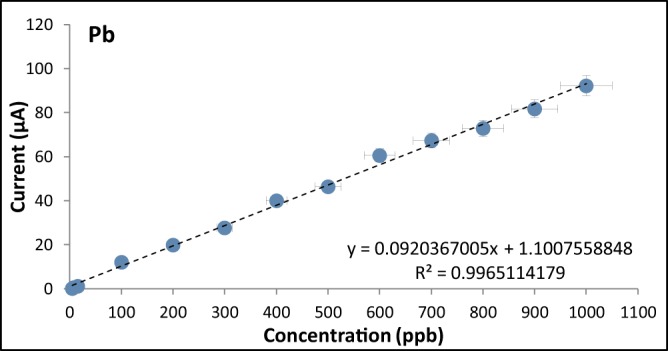
Calibration curve of the optimized modified GE for Pb.

The calibration curves were obtained by varying the analyte concentrations from 5 ppb to 1000 ppb for both cadmium and lead (Figs. 12, 13, 14). The R^2^ values were found to be 0.9968 for Cd and 0.9965 for Pb indicating a strong linear relationship between the heavy metal concentration and the anodic current peak.

**Figure 15:**
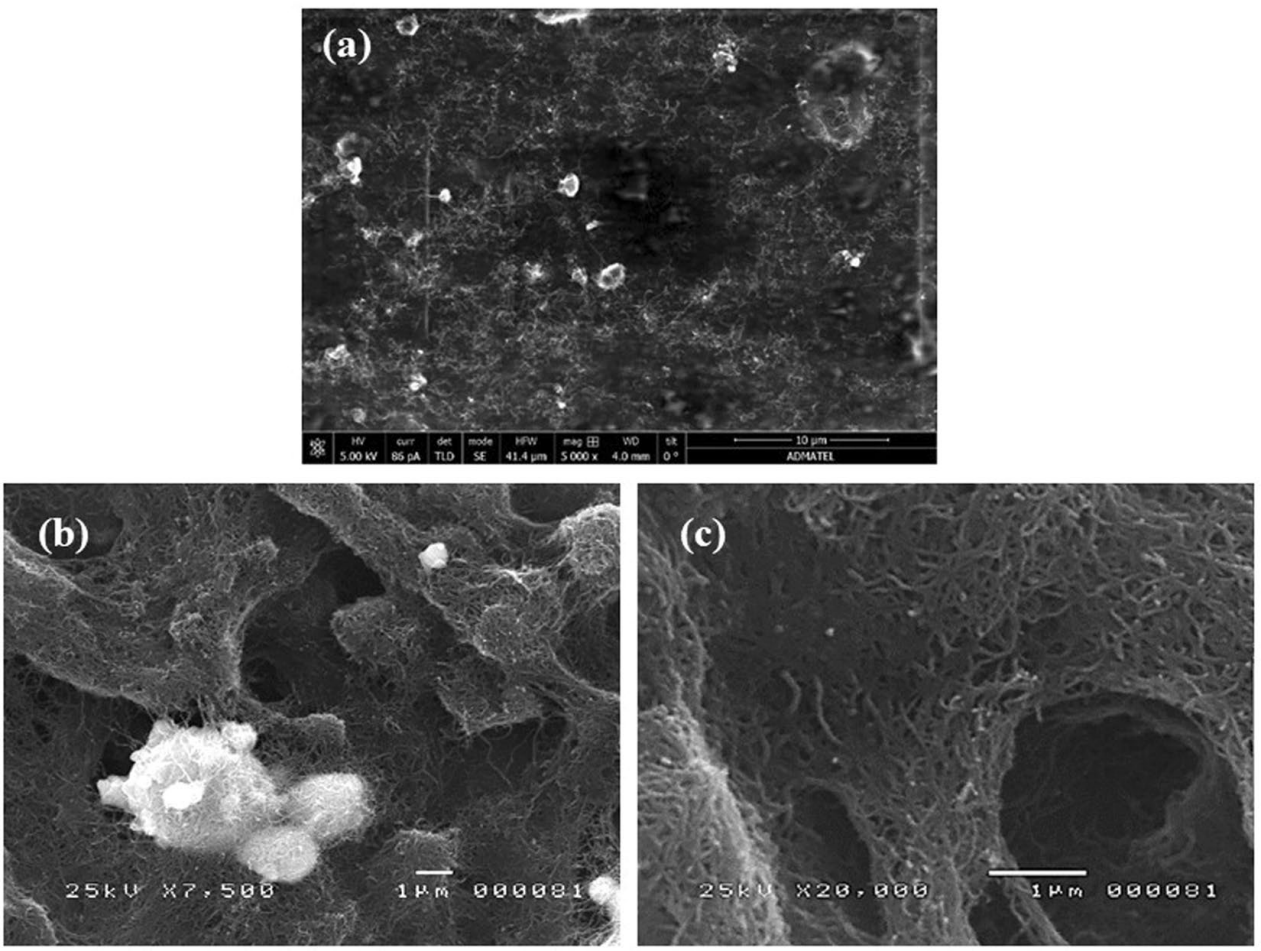
SEM micrographs of the optimized modified GE (**a**) at 5,000x magnification, (**b**) at 7,500x magnification, and (**c**) at 20,000x magnification.

### Characterization of the optimized modified graphite electrode

#### Surface morphology analysis

**Figure 16:**
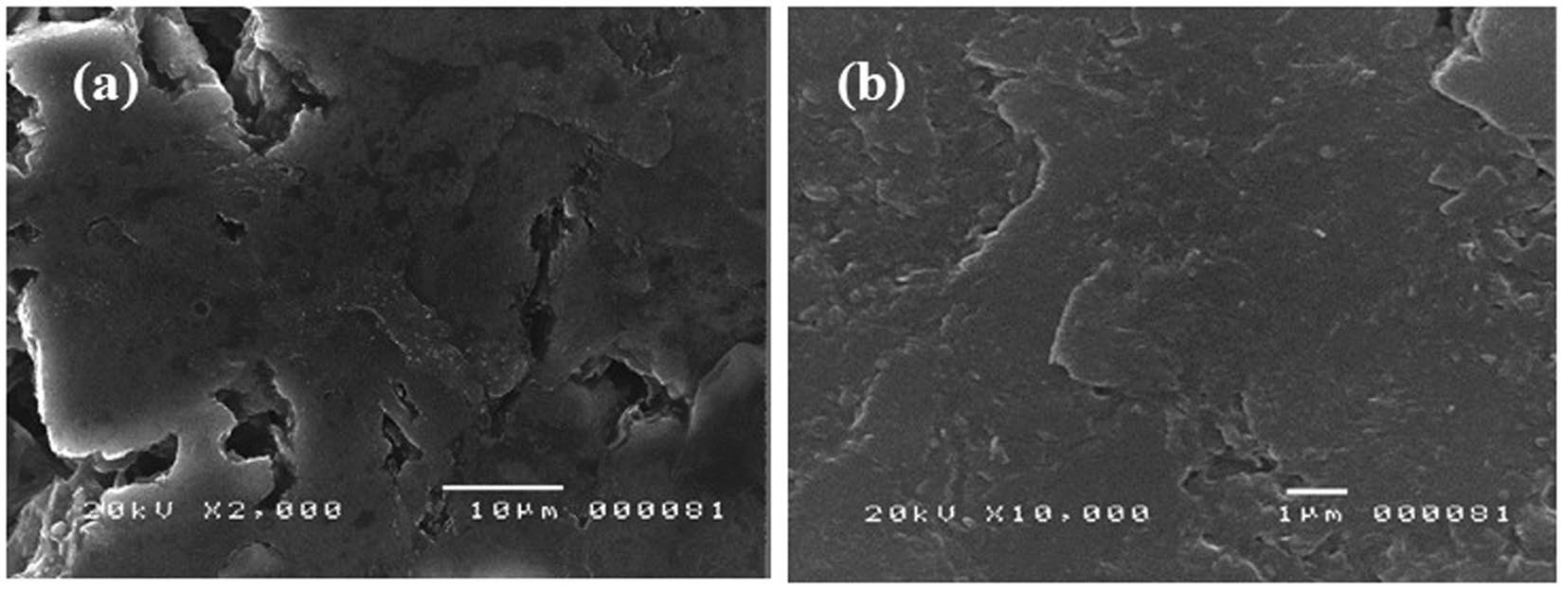
SEM micrographs of the bare GE (**a**) 2,000x magnification and (**b**) at 10,000x magnification.

The modified electrode’s surface morphology was studied with the use of a scanning electron microscope (SEM). Figure 15 shows the successful deposition of a web-like structure of MWCNT on the electrode’s surface and the MWCNT matrix’s ability to act as a binding agent for the BiNPs. In addition, the uniform coating of dispersed bead-shaped BiNPs can be seen in Fig. 15b,c. For comparison, the surface morphology of the bare graphite electrode was studied at 10,000 magnification. Figure 16 shows the bare graphite electrode’s surface with the natural flaky morphology of the graphite rod but **w**ithout the presence of BiNPs and MWCNTs.

#### Elemental analysis and mapping

**Figure 17:**
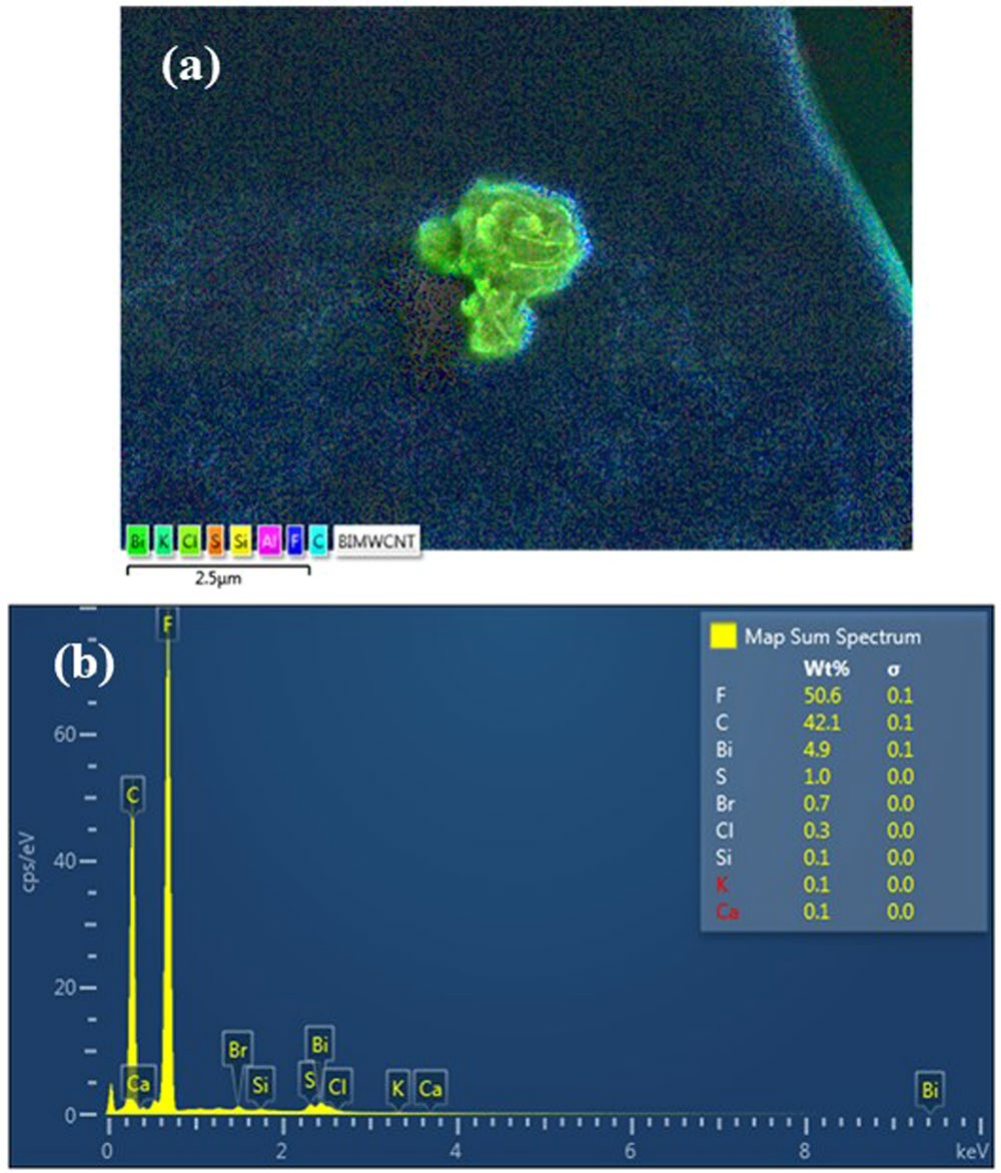
(**a**) Elemental mapping and (**b**) EDX spectrum of the optimized modified GE.

The modified electrode was subjected to Energy Dispersive X-ray (EDX) analysis to confirm the elemental compositions of the modifiers. Figure 17 shows the elemental mapping and the corresponding EDX spectrum of the optimized modified GE. Both the map and the spectrum confirm the presence of the modifiers on the electrode surface. The high weight percentage of fluorine (F) is due to the presence of Nafion while the high amount of carbon (C) is due to the MWCNTs and the graphite electrode itself. The bead-shaped particles in the SEM micrographs were indeed BiNPs as revealed by the elemental mapping and the presence of bismuth in the EDX spectrum.

### Real sample analysis

Real sample analysis via ASV was done using the optimized modified graphite electrode and optimum ASV parameters. The concentrations of heavy metals in the real samples were determined by substituting the values of the current peak as the y-values in the equations from the calibration curves of Cd and Pb. Atomic absorption spectrometry (AAS) analysis of the real samples was carried out to verify the results obtained from ASV. The concentrations of the heavy metals in the real samples were determined by substituting the values of the absorbance as the y-values in the equations from the calibration curves of Cd and Pb.

The results of real sample analysis via ASV and AAS were compared by computing the mean and standard deviation (Table 1). The detected concentrations of Cd from the voltammetric analysis were very similar to the results from AAS except for the *Guyabano Herbal Tea*. Due to the insignificant difference in the detected concentrations of Cd between the two methods, the fabricated modified electrode is proven to be as reliable as AAS for the detection of Cd. However, the concentrations of lead in the samples were not as close as compared to cadmium. This was probably due to the inhomogeneity of the assay when the AAS measurements were taken. Nevertheless, the voltammetric results of the analysis of the real samples prove the advantage of ASV as compared to AAS since it was able to detect other heavy metals from the real samples without prior knowledge of the presence of heavy metals in the sample, i.e., Cu ions were detected in the real samples. While in AAS, the heavy metals present in the sample must be known before they are detected and quantified.

**Table 1:** Comparison of Detected Concentrations of Cd and Pb in real samples.

Sample Name	CADMIUM		LEAD	
	ASV (ppb)	AAS (ppb)	ASV (ppb)	AAS (ppb)
Banaba	244.50 ± 1.86	234.85 ± 0.87	16.88 ± 0.63	473.43 ± 1.40
Bignay	230.17 ± 1.69	238.52 ± 0.68	9.29 ± 0.62	521.29 ± 6.40
Guyabano	109.39 ± 2.50	263.70 ± 1.40	235.91 ± 8.17	611.87 ± 12.17

### Limits of detection

The limit of detection (LOD) of the bare electrode was found to be 115.37 ppb for Cd^2+^ and 112.10 ppb for Pb^2+^ while that of the modified electrode was found to be 1.06 ppb for Cd^2+^ and 0.72 ppb for Pb^2+^. The LOD of the fabricated bare and modified graphite electrode in this study were compared with similar electrodes from previous works in literature as shown in Table 2. The obtained detection limits of the fabricated modified electrode for both heavy metals were superior to most of the previous works, much more when the difference in the purity of the electrodes are taken into consideration since the graphite rod was not originally made as a sensor unlike the analytical grade electrodes such as the GCE.

**Table 2:** Performance comparison of the bare and modified GE with other works.

Electrode	Modifiers	Method	Heavy Metals Detected	LOD		Ref.
				Cadmium	Lead	
Glassy Carbon Electrode	Crosslinked Chitosan and Carbon Nanotubes Film	SWASV	Cd, Pb, and Cu	800 ppb	600 ppb	^66^
Carbon Paste Electrode	Coconut Shell Powder	ASV	Cd	105 ppb	—	^67^
Pencil Graphite Electrode	Bismuth Film	ASV	Cd and Pb	11 ppb	11.5 ppb	^68^
Pencil Graphite Electrode	—	DPV	Pb	—	8.9 ppm	^69^
Glassy Carbon Electrode	Tris (2,2′-bipyridyl) ruthenium(II), Graphene, and Nafion	DPV	Cd, Pb, and Cu	49 ppb	48 ppb	^70^
Glassy Carbon Electrode	Gold Nanoparticles, Hexaammineruthenium, and Nafion	ASV	Cd and Pb	45 ppb	200 ppb	^71^
Indium Tin Oxide Electrode	[Ru (NH_3_)^6^]^3+^ and Nafion	ASV	Cd, Pb, and Zn	500 ppb	500 ppb	^72^
Graphene Paste Electrode	Coconut Husk Activated Carbon	ASV	Cd and Pb	56 ppb	44 ppb	^73^
Graphene Paste Electrode	Silver Nanoparticles	ASV	Cd, Pb, and Cu	17 ppb	12 ppb	^74^
Battery Graphite Electrode	—	ASV	Cd and Pb	115.37 ppb	112.1 ppb	This work
Battery Graphite Electrode	Bismuth, MWCNT, and Nafion		Cd, Pb, and Cu	1.06 ppb	0.72 ppb	

## Conclusions

Graphite rods from waste zinc-carbon batteries were modified with bismuth, multi-walled carbon nanotubes, and Nafion and were successfully used to detect cadmium and lead ions via ASV. Among the five brands used for detection, the *Eveready Heavy Duty* exhibited the highest anodic current peaks for both heavy metals. The drop-coating method used in the study was successful in modifying the surface of the bare electrode with BiNPs, MWCNTs, and Nafion as evidenced by the SEM micrographs and EDX analysis results. Among the nine combinations of modifiers, 1 mg BiNPs and 1 mg MWCNTs mixed in a 5 mL Nafion-ethanol solution resulted with the highest anodic current peaks for Cd and Pb. Modification of the electrode surface remarkably enhanced the voltammetric response of the heavy metals which can be attributed to the synergetic effects of BiNP, MWCNT and Nafion. The calibration curves of the bare electrode exhibited excellent linearity from 100 ppb to 1000 ppb, while the modified electrode showed excellent linearity from 5 ppb to 1000 ppb. This, in turn, gave low detection limits (115.37 ppb and 112.1 ppb for cadmium and lead, respectively, for the bare electrode and 1.06 ppb and 0.72 ppb for cadmium and lead, respectively for the modified electrode) which are the desired figures of merit. The modified graphite electrode was successfully utilized for the detection of Cd^2+^ and Pb^2+^ in herbal food supplement samples with good agreement to the results obtained by atomic absorption spectroscopy. Thus, the electrochemical sensor developed in this study is not only cost-effective but environment-friendly as well. Modification of the graphite electrode with other materials and its capability of detecting species other than heavy metals may be further explored.

## Data Availability

The datasets generated during and/or analysed during the current study are available from the corresponding author on reasonable request.
